# Virologic outcomes of second-line antiretroviral therapy in Eastern European country of Georgia

**DOI:** 10.1186/1742-6405-11-18

**Published:** 2014-07-07

**Authors:** Nikoloz Chkhartishvili, Lali Sharvadze, Natia Dvali, Marine Karchava, Nino Rukhadze, Maia Lomtadze, Otar Chokoshvili, Tengiz Tsertsvadze

**Affiliations:** 1 Infectious Diseases, AIDS and Clinical Immunology Research Center, 16 Al. Kazbegi Avenue, Tbilisi 0160, Georgia; 2 I. Javakhishvili Tbilisi State University Faculty of Medicine, 16 Al. Kazbegi Avenue, Tbilisi 0160, Georgia

**Keywords:** HIV, Antiretroviral therapy, Second-line ART, Eastern Europe

## Abstract

**Background:**

Data on the effectiveness of second-line antiretroviral therapy (ART) in resource-limited countries of Eastern Europe is limited. Objective of this study was to evaluate virological outcomes of second-line ART in Georgia.

**Methods:**

We conducted retrospective analysis using routinely available program data. Study included adult HIV-infected patients with confirmed HIV drug resistance, who were switched to second-line ART from August 2005 to December 2010. Patients were followed until July 1, 2011. Primary outcome was achievement of viral suppression. Demographic, clinical, laboratory and adherence data were abstracted from medical and program records. Adherence was expressed as percentage based on medication refill data, and was calculated as days supply of medications dispensed divided by days between prescription fills. Predictors of primary outcome were assessed in modified Poisson regression analysis.

**Results:**

A total of 84 patients were included in the study. Among them 71.4% were men and 62% had history of IDU. All patients were receiving non-nucleoside reverse transcriptase based regimen as initial ART. The mean 6-month adherence prior to virologic failure was 75%, with 31% of patients showing 100% adherence. All patients were switched to protease inhibitor based regimens. Patients were followed for median 27 months. Over this period 9 (10.7%) patients died. Among 80 patients remaining alive at least 6 month after ART regimen switch, 72 (90%) patients ever reached undetectable viral load. The mean first 6-month adherence on second-line treatment was 81%, with 47.5% of patients showing 100% adherence. The proportion of patients achieving viral suppression after 6, 12, 24 and 36 months of second-line ART did not vary significantly ranging from 79 to 83%. Percentage of IDUs achieving viral suppression ranged from 75% and 83%. Factors associated with failure to achieve viral suppression at 6-months of second-line ART were: adherence <80% (Risk ratio [RR] 5.09, 95% CI: 1.89-13.70) and viral load >100,000 at the time of treatment failure (RR 3.39, 95% CI: 1.46-7.89).

**Conclusions:**

The study demonstrated favourable virological outcomes of the second-line ART in Georgia. Majority of patients, including IDUs, achieved sustained virological response over 36 month period. The findings highlight the need of improving adherence.

## Background

The number of people receiving antiretroviral therapy (ART) in resource limited settings is climbing rapidly. At the end of 2012 estimated 9.7 million people in low- and middle-income countries had access to ART, representing 40-fold increase over the last decade [1]. As scale-up of ART continues growing number of patients experience treatment failure increasing demand on second-line regimens [2-4].

There is limited data on the use of second-line ART in Eastern European region, where HIV epidemic is primarily driven by injection drug users (IDU). Although region has low ART coverage, the absolute number of persons accessing the lifesaving therapy has been annually increasing and reached nearly 200,000 in 2012 [1]. Better understanding of second-line ART delivery in the region will be important to ensure its long-term effectiveness.

Georgia is an Eastern European nation located in the Caucasus between Turkey and Russia. Similar to other countries in the region the HIV epidemic in Georgia has been concentrated around IDUs, accounting for 55% of total reported cases of HIV infection. Since 2004, through the support from the Global Fund to Fight AIDS, Tuberculosis and Malaria Georgia has ensured universal access to ART. UNAIDS estimates conform that Georgia has the highest ART coverage in the region [1]. Previously we showed that ART substantially reduced mortality and increased survival in Georgia [5,6]. The objective of the current study was to evaluate virologic outcomes of second-line ART in the country.

## Methods

We conducted retrospective analysis using routinely available program data. Study included adult (age ≥18 years) HIV-infected patients enrolled in national HIV/AIDS treatment and care program. Patients with confirmed HIV drug resistance, who were switched to second-line ART regimens from August 2005 to December 2010 were included in the analysis. Patients were followed until July 1, 2011.

Georgia’s national HIV/AIDS treatment and care program provides HIV related medical services to all diagnosed HIV patients. The program is coordinated by the Infectious Diseases, AIDS and Clinical Immunology Research Center (IDACIRC), which is country’s referral institution for HIV diagnosis, treatment and care. Patients receive services at IDACIRC clinic and three dedicated regional facilities.

Provision of ART in Georgia is governed by national HIV/AIDS treatment and care guidelines, which was first developed in 2004. During the study period (2005–2010) ART was recommended at CD4 count ≤200 cells/mm^3^. The recommended initial regimen consisted of two nucleoside reverse transcriptase inhibitors (NRTI) and one non-nucleoside reverse transcriptase inhibitor (NNRTI). Zidvudine (AZT) + lamivudine (3TC) has been preferred NRTI component since 2004. Stavudine (d4T) was phased out after 2007 revision of national guidelines, and was replaced by abacavir (ABC) + lamivudine (3TC) or tenofovir (TDF) + emtricitabine (FTC). Efavirenz (EFV) has been the preferred NNRTI with nevirapine (NVP) being recommended as an alternative to EFV.

According to national guidelines follow-up of patients on ART relied on regular monitoring of CD4 count and HIV viral load. HIV drug resistance was performed routinely among patients with virologic failure, defined as confirmed plasma HIV RNA >400 copies/ml 6 months after starting therapy or after undetectable viral load while on therapy. Results of drug résistance testing were used for selection of second-line therapy.

During the study period plasma HIV RNA levels were measured using either Amplicor HIV-1 Monitor test, version 1.5 (Roche Molecular Diagnostics, Germany) or the real-time PCR assay COBAS TaqMan HIV-1 test (Roche Molecular Diagnostics, Germany). For genotypic resistance testing the TruGene HIV-1 Genotyping Kit was employed according to the manufacturer’s instructions using OpenGene DNA Sequencing System (Siemens Medical Solutions Diagnostics, Germany). The Stanford University algorithm (http://hivdb.stanford.edu/) were used for resistance interpretation.

Demographic, epidemiological, clinical and laboratory data were extracted from program and medical records. Baseline characteristics were collected at the time point when patient experienced virologic failure. Adherence was expressed as percentage based on medication refill data, and was calculated as days supply of medications dispensed divided by days between prescription fills. The primary outcome was proportion of patients achieving viral suppression (<400 copies/ml) after the switch to second line ART. Predictors of primary outcome were assessed in modified Poisson regression analysis [7]. All statistical analyses were performed using SAS 9.2.

Study was approved by the Institutional Review Board of IDACIRC.

## Results

Table 1 summarizes characteristics of 84 patients included in the analysis. Among them the majority were men (71.4%) and 62% had history of IDU. At the time of virologic failure on initial ART, all patients were receiving NNRTI based regimen, and vast majority (77%) were on EFV (77%). AZT + 3TC was the most common NRTI backbone, prescribed to 63% of patients. The mean 6-month medication refill adherence prior to virologic failure was 75%, with 31% of patients timely picking-up their prescription. None of the patients had virus with resistance mutations to PIs, and 86% had NRTI/NNRTI dual class-resistant viruses. All patients were switched to PI-containing regimens, including 62 (73.8%) to ritonavir boosted Lopinvair (LPV/r)-based and 22 (26.2%) to ritonavir boosted Atazanavir (ATV/r)-based regimens.

**Table 1:** Patient characteristics at the time of virological failure on initial ART (n = 84)

	**n = 84**
**Age, median years (IQR)**	37 (34–44)
**Sex, n (%)**	
Men	60 (71.4)
Women	24 (28.6)
**Mode of HIV transmission, n (%)**	
Injection drug use	52 (61.9)
Heterosexual contact	28 (33.3)
Male-to-male sex	2 (2.4)
Blood recipient	2 (2.4)
**HCV infection, n (%)**	
anti-HCV+	45 (53.6)
anti-HCV-	39 (46.4)
**Tuberculosis, n (%)**	
History of TB	35 (41.7)
No history of TB	49 (58.3)
**ART regimen at failure, n (%)**	
Zidovudine + Lamivudine + Efavirenz	39 (46.4)
Zidovudine + Lamivudine + Nevirapine	14 (16.7)
Abacavir + Lamivudine + Efavirenz	17 (20.2)
Abacavir + Lamivudine + Nevirapine	3 (3.6)
Stavudine + Lamivudine + Efavirenz	7 (8.3)
Stavudine + Lamivudine + Nevirapine	2 (2.4)
Tenofovir + Emtricitabine + Efavirenz	2 (2.4)
**6-month adherence prior to failure, n (%)**	
<80%	36 (42.9)
80- < 100%	22 (26.2)
100%	26 (30.9)
**Time to failure, median (IQR)**	18 (12–34)
**Viral load at failure, median copies/ml (IQR)**	23600 (8365–126000)
**Number of resistant mutations, median (IQR)**	2 (1–4)
**Drug class resistance, n (%)**	
Single class (NRTI or NNRTI)	12 (14.3)
Dual class (NRTI + NNRTI)	72 (85.7)

**Figure 1:**
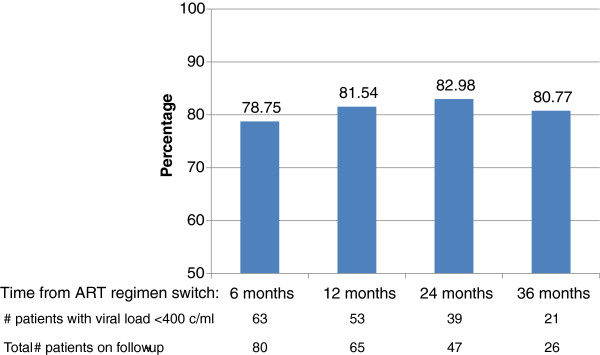
The proportion of patients achieving viral suppression after initiating second-line antiretroviral therapy.

Patients were followed for median 27 (Interquartile range [IQR]: 13–41) months. Over this period 9 (10.7%) patients died in median 6 (IQR: 2–8) months after treatment switch, four of them died within 6 month period.Among 80 patients remaining alive at least 6 month after ART regimen switch, 72 (90%) patients ever achieved viral suppression after median 7 (IQR: 5–10) months. The mean first 6-month adherence on second-line treatment was 81%, with 47.5% of patients showing 100% adherence. The proportion of patients achieving viral suppression after 6, 12, 24 and 36 months of second-line ART did not vary significantly ranging from 79 to 83% (Figure 1). Percentage of IDUs achieving viral suppression ranged from 75% and 83%. During the follow-up five patients met the criteria of virologic failure and were tested for HIV drug resistance. None of the strains evaluated carried major PI mutations.

Factors associated with failure to achieve viral suppression after 6 months of regimen modification were evaluated in multivariate analysis. Lower adherence <80% (Risk ratio [RR] 5.09, 95% Confidence interval [CI]: 1.89-13.70) and viral load of greater than 100,000 at the time of treatment failure (RR 3.39, 95% CI: 1.46-7.89) were significantly associated with detectable viral load (Table 2). Neither gender nor mode of HIV transmission showed association with the outcome.

**Table 2:** Factors associated with failure to achieve viral suppression 6 months after switching ART regimen (n = 80)

	**Total N**	**Detectable viral load**		
		**n (%)**	**Bivariate**	**Multivariate**
			**RR (95% CI)**	**RR (95% CI)**
**Age**				
<37 years	35	7 (20.0)	0.90 (0.38-2.12)	
≥37 years	45	10 (22.2)	1	
**Sex**				
Men	57	13 (22.8)	1.31 (0.48-3.60)	0.50 (0.17-1.45)
Women	23	4 (17.4)	1	1
**Mode of HIV transmission**				
IDU	49	12 (24.5)	1.52 (0.59-3.89)	1.34 (0.64-2.83)
non-IDU	31	5 (16.1)	1	1
**HCV infection**				
anti-HCV+	42	9 (21.4)	1.02 (0.44-2.37)	
anti-HCV-	38	8 (21.1)	1	
**Tuberculosis**				
History of TB	31	6 (19.4)	0.86 (0.36-2.09)	
No history of TB	49	11 (22.5)	1	
**Viral load at failure on initial ART**				
≥100,000	21	9 (42.9)	3.16 (1.40-7.12)	3.39 (1.46-7.89)
<100,000	59	8 (13.6)	1	1
**ART regimen after switch**				
ATV/r-based	20	4 (20.0)	0.92 (0.34-2.51)	
LPV/r-based	60	13 (21.7)	1	
**6-month adherence after regimen switch**				
<80%	19	10 (52.6)	5.01 (1.80-13.87)	5.09 (1.89-13.70)
80- < 100%	23	3 (13.0)	1.24 (0.30-5.05)	1.48 (0.36-6.12)
100%	38	4 (10.5)	1	1
**Number of resistance mutations**				
>2 mutations	21	3 (14.3)	0.60 (0.19-1.89)	
≤2 mutations	59	14 (23.7)	1	
**Drug class resistance**				
Single class (NRTI or NNRTI)	11	3 (27.3)	1.34 (0.46-3.93)	
Dual class (NRTI + NNRTI)	69	14 (20.3)	1	

## Discussion

Findings of this analysis indicate that majority of patients with HIV drug resistance in Georgia achieve and maintain viral suppression after switching to second-line therapy. To the best of our knowledge this is the first published study that evaluated outcomes of second-line ART in Eastern Europe, therefore regional comparisons cannot be made. Early virologic outcomes seen in our study were generally consistent with findings from other resource-limited countries, primarily from Africa and Asia, with approximately 80% of patients on second-line ART achieving viral suppression at 6 and 12 months [8-12]. Recent meta-analysis of 19 studies found that that proportion of patients achieving viral suppression dropped from 78% at 6 months to 62% at 36 months of starting second-line regimen [8]. However, there was substantially heterogeneity between studies, with some studies reporting success rates of around and more than 90% [13-16]. In our study more than 80% of patients consistently had undetectable levels of viral load after 6 months of second-line ART.

Multivariate analysis showed that adherence of <80% was the strongest predictor of failure to achieve viral suppression after 6 months of second-line ART (RR 5.09, 95% CI: 1.89-13.70). There was no statistically significant difference between adherence levels of 100% and 80- < 100%. Also, none of five patients screened for HIV drug resistance after failure of second-line ART harbored the viruses with major PI mutations. These data confirm that ritonavir-boosted PI regimens in Georgian settings are ‘forgiving’ as seen elsewhere [17] and that non-adherence is the primary reason of virologic failure, but not resistance. Similar findings were reported by Murphy and colleagues [10]. Although overall adherence improved after treatment modification, efforts are still needed to ensure high levels of adherence in a long term in all patients.

The reported rates of mortality among patients on second-line ART varied substantially, ranging from 3% to 16% [18-21]. In our study 11% of patients died over the follow-up, including four patients died within 6 months after the switch. It is unlikely that mortality in our study was directly related to virologic failure to initial regimen. Routine use of viral load in Georgia and low number of drug resistance mutation at the time failure indicates that regimens were modified timely. Analysis of causes of death also does not support linkage between virologic failure and mortality in our study. Six (67%) of 9 deaths reported were attributable to non-AIDS related causes, including end stage liver disease and cardiovascular disease.

Our study provides evidence that persons with history of IDU can achieve optimal treatment outcomes. Percentage of IDUs achieving viral suppression ranged from 75% and 83% after initial treatment modification. Also, history of IDU was not associated with virologic failure in multivariate analysis. This has important implications for the Eastern European region, where IDUs are often face barriers for accessing ART [22]. Our study clearly shows that IDUs can achieve optimal treatment outcomes and that no one should be withheld from therapy. Factors that might have contributed to optimal outcomes among persons with history of IDU include environment with free HIV-related medical care, availability of adherence support services and methadone substation therapy.

Our study is subject to limitations. First this is the small sample size, which limited statistical power and generalizability of our findings. Also, we conducted retrospective analysis utilizing routinely available data, thus there might have been unmeasured factors that influenced the outcomes. We only had information on history of IDU, based on HIV transmission category. Information on ongoing drug abuse may have influenced comparisons. Viral load was measured by physician’s discretion and we extracted measurements closest to the time intervals studied.

## Conclusions

In conclusion, our study shows that second-line therapy is effective in IDU-driven epidemic setting of Georgia. More than 80% of patients, including those with history of IDU, maintained viral suppression over 36 months period. Although adherence improved after switching to second-line ART, it showed to be the most important factor associated with virologic outcome. Efforts are needed to ensure durability of adherence and hence viral suppression. Persons with history of IDU can fully benefit from second-line ART.

## Abbreviations

ART: Antiretroviral therapy; HIV: Human immunodeficiency virus; AIDS: Acquired immune deficiency syndrome; IDU: Injection drug use; NRTI: Nucleoside reverse transcriptase inhibitor; NNRTI: Non-nucleoside reverse transcriptase inhibitor; PI: Protease inhibitor; IQR: Interquartile range; ATV/r: Atazanavir/ritonavir; LPV/r: Lopinavir/ritonavir; IDACIRC: Infectious Diseases AIDS and Clinical Immunology Research Center.

## Competing interests

The authors declare that they have no competing interest.

## Authors’ contributions

NC conducted statistical analysis and prepared the first draft of the manuscript. LS and TT contributed to concept and study design, data interpretation and also critically reviewed the manuscript. ND, MK, NR and ML contributed to data acquisition and edited the manuscript. OC prepared study datasets and contributed to data analysis. All authors read and approved the final manuscript.
